# Variance and Scale-Free Properties of Resting-State Blood Oxygenation Level-Dependent Signal After Fear Memory Acquisition and Extinction

**DOI:** 10.3389/fnhum.2020.509075

**Published:** 2020-10-09

**Authors:** Alina Tetereva, Sergey Kartashov, Alexey Ivanitsky, Olga Martynova

**Affiliations:** ^1^Institute of Higher Nervous Activity and Neurophysiology of the Russian Academy of Sciences, Moscow, Russia; ^2^Department of Psychology, University of Otago, Dunedin, New Zealand; ^3^National Research Centre Kurchatov Institute, Moscow, Russia; ^4^Centre for Cognition and Decision Making, Institute of Cognitive Neuroscience, National Research University Higher School of Economics, Moscow, Russia

**Keywords:** scale-free dynamics, BOLD signal, fear extinction, variance, hurst exponent, detrended fluctuation analysis, fMRI

## Abstract

Recently, the dynamic properties of brain activity rather than its stationary values have attracted more interest in clinical applications. It has been shown that brain signals exhibit scale-free dynamics or long-range temporal correlations (LRTC) that differ between rest and cognitive tasks in healthy controls and clinical groups. Little is known about how fear-inducing tasks may influence dispersion and the LRTC of subsequent resting-state brain activity. In this study, we aimed to explore the changes in the variance and scale-free properties of the brain’s blood oxygenation level-dependent (BOLD) signal during the resting-state sessions before and after fear learning and fear memory extinction. During a 1-h break between magnetic resonance imaging (MRI) scanning, 23 healthy, right-handed volunteers experienced a fear extinction procedure, followed by Pavlovian fear conditioning that included partial reinforcement using mild electrical stimulation. We extracted the average time course of the BOLD signal from 245 regions of interest (ROIs) taken from the resting-state functional atlas. The variance of the BOLD signal and the Hurst exponent (*H*), which reflects the scale-free dynamic, were compared in the resting states before and after fear learning and fear memory extinction. After fear extinction, six ROIs showed a difference in *H* at the uncorrected level of significance, including areas associated with fear processing. *H* decreased during fear extinction but then became higher than before fear learning, specifically in areas related to the fear extinction network (FEN). However, activity in the other ROIs restored the *H* to its initial level. The variance of the BOLD signal in six ROIs demonstrated a significant increase from initial rest to the post-task rest. A limited number of ROIs showed changes in both *H* and variance. Our results imply that the variability and scale-free properties of the BOLD signal might serve as additional indicators of changes in spontaneous brain activity related to recent experience.

## Introduction

In recent years, research on the human brain demonstrates an increasing interest to the dispersion and dynamic properties of brain activity than to its stationary values. Fluctuations are essential for maintaining the optimal state of brain activity and for flexibility between states (McIntosh et al., 2010; Tognoli and Kelso, 2014). In functional neuroimaging studies, the variability of brain dynamics can be most simply described through the variance (σ^2^) of the blood oxygenation level-dependent (BOLD) signal. This variance provides additional information about the signal change in comparison to conventional measures as the averaged activity. Several works have shown strong associations of the BOLD signal variance with age (Garrett et al., 2011, 2013; Nomi et al., 2017), task performance (He, 2011; Garrett et al., 2012), and performance efficiency (Burzynska et al., 2015). The variance also differs with mental diseases, including autism (Di Martino et al., 2014), Alzheimer’s disease (Zhao et al., 2015), and schizophrenia (Yu et al., 2014).

A pattern of fluctuations or neural dynamics can be assessed not only via the standard deviation of measured parameters but also via the fractal properties of the brain signal. While temporal variance depends on the size of time window, a multi-scale approach measuring fractality considers variance across different window sizes. Fractality is a quite frequent property in nature, demonstrating self-similarity from micro- to macro-structures (Bassingthwaighte et al., 1994). It can be found not only in the spatial structure of some objects but also in the temporal behavior of systems. The human brain is no exception. Brain activity shows behavior that is fractal (also called self-similar or scale-free), meaning that no characteristic scales dominate the neural dynamics (Linkenkaer-Hansen et al., 2001; Bullmore et al., 2009). Such behavior manifests in the long-range temporal correlations (LRTC) of the signal and 1/*f*-like spectral power *P*(*f*) → 1/*f*^β^, where *P* is power, *f* is frequency, and β is called the “power-law exponent” (Bullmore et al., 2001; Bullock et al., 2003).

The LRTC can be parameterized using the Hurst exponent (*H*), which is expressed as β = 2*H*−1 regarding the power-law exponent for fractional Brownian noise model (Hurst, 1951). A larger *H* value indicates a more auto-correlated signal. The value of *H* for a stationary signal can range from 0 to 1. Values larger than 1 indicate a non-stationary process, and *H* calculates as *H* = α−1. Signal series, depending on the index, can be divided into three categories. First, for 0 < *H* < 0.5, the series are anticorrelated. Second, *H* = 0.5 is a property of random noise. Third, 0.5 < *H* < 1 indicates the fractal complexity of the signal and the presence of LRTC (Hardstone et al., 2012).

Beginning with a study by Linkenkaer-Hansen et al. (2001), LRTC were actively explored in EEGs, which showed the scale-free behavior of brain oscillations in various frequencies. The fractal pattern was also found in the temporal dynamics of the BOLD signal (Zarahn et al., 1997; Bullmore et al., 2001). Initially, it was assumed that self-similar fluctuations in functional magnetic resonance imaging (fMRI) signals could be pink noise from the operation of the equipment (Zarahn et al., 1997). Later, more detailed studies provided strong evidence that scale-free dynamics is an intrinsic property of the signal (He et al., 2010). Remarkably, different brain tissues produce different dynamics: the power-law exponent varies between the gray and white matter and the cerebrovascular fluid (Bullmore et al., 2004), as well as between different functional networks (He et al., 2010).

The LRTC in specific brain areas may change with conditions. *H* decreased during task performance in comparison to during the resting state (He, 2011; Ciuciu et al., 2012), suggesting that resting-state neural activity exhibits more long-term memory. Furthermore, the LRTC altered with healthy aging (Suckling et al., 2008; Dong et al., 2018); personal traits, including impulsivity (Hahn et al., 2012; Akhrif et al., 2018); and mental diseases, including major depressive disorder (Wei et al., 2013), schizophrenia (Sokunbi et al., 2014), and Alzheimer’s disease (Maxim et al., 2005). The increased temporal variance was also shown to reflect system functioning efficiency as it positively correlated with an increase of functional connectivity depending on higher cognitive processing (Garrett et al., 2012, 2018). However, little is known about how fear-inducing conditions may influence the variance and fractality of brain activity during the task and subsequent resting state. Previous studies reported that stress and fear could induce temporal changes in the resting-state functional connectivity (Schultz et al., 2012; Feng et al., 2015; Martynova et al., 2020). Abnormal resting-state connectivity was also repeatedly shown in fear-conditioning studies in patients who had post-traumatic stress disorders (PTSD) (Rauch et al., 2006; Rabinak et al., 2011) and anxiety (for review, Northoff, 2020). Few papers have shown a positive relation of anxiety scores and the BOLD signal LRTC of brain regions involved in emotional and cognitive processing (Tolkunov et al., 2010; Churchill et al., 2015). The increase of the amygdala’s temporal variance was found to be associated with state anxiety and functional connectivity of the BOLD signal during acute systemic inflammation (Labrenz et al., 2019). Nevertheless, to our knowledge, there were no direct studies of task-based and resting-state fMRI fractality and variance behavior induced by fear conditioning paradigms.

Based on our previous findings on persistent changes of functional connectivity in resting state after fear learning and fear memory extinction (Tetereva et al., 2020; Martynova et al., 2020), we assumed that if a regional and network correlation of the brain activity can change after stimulation, it should consequently be reflected in the variance and scale-free properties of the BOLD signal. To test this hypothesis, we tested changes that occurred in the LRTC of resting-state BOLD signals after fear learning and fear memory extinction in comparison to the initial pre-task resting state. A similar design comparing “rest–task–rest” activity has been applied in only a few fMRI studies. One focused on the spectral density of the BOLD response (Duff et al., 2008). Another used working-memory tasks to investigate the fractal properties of the brain dynamics in the rest–task–rest paradigm (Barnes et al., 2009). We hypothesized that short exposure to emotionally negative stimulation during Pavlovian fear conditioning might change neural efficiency and corresponding variance and scaling exponent of the BOLD signal in the subsequent resting state in the specific regions related to fear processing. Therefore, we expected a decrease of the scaling exponent during fear extinction in the fear-processing brain network, similar to the findings on its suppression with cognitive load (Barnes et al., 2009; Churchill et al., 2016). Simultaneously, we hypothesized that the variance should increase during the task as an indirect index of neural efficiency (Labrenz et al., 2019). We also expected to find residual changes of both LRTC and variance in areas of fear-processing brain network in the post-task resting state, as it was early reported for functional connectivity of the BOLD signal (Schultz et al., 2012; Feng et al., 2015; Martynova et al., 2020). Understanding the variability and fractal properties of brain dynamics may enhance knowledge regarding the spatiotemporal structure of spontaneous brain activity, its modulation after emotional stimulation, and its potential as a biomarker of affective disorders.

## Materials and Methods

### Participants

Twenty-seven volunteers participated in the fMRI study. None had histories of psychiatric or neurological disorders, and all had a normal or corrected-to-normal vision. Functional imaging data of four participants with excessive averaged displacement and motion spikes bigger than voxel size were excluded from the analysis. The final study sample included 23 healthy, right-handed volunteers (23.90 ± 3.93 years old, 8 females). In addition, participants completed the State-Trait Anxiety Inventory (STAI) before scanning (state: 33.70 ± 10.95; trait: 41.22 ± 12.87). In Russian adaptation (Khanin, 1976), the score may vary from 20 to 80 points. The score below 30 points means low anxiety level, and above 46 means very high. The scores in interval 31–45 mean a moderate level. The study’s protocol followed the requirements of the Helsinki Declaration, and the study was approved by the Ethics Committee of the Institute of Higher Nervous Activity and Neurophysiology of the Russian Academy of Science. All subjects provided written, informed consent before the study.

### Procedure

**FIGURE 1:**
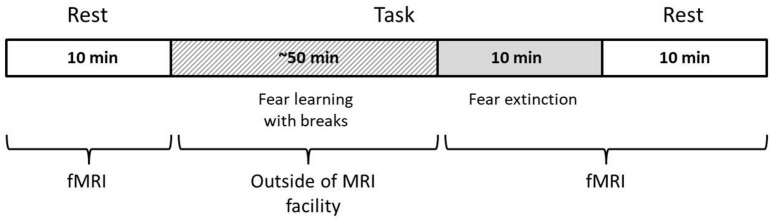
Scheme of the experimental paradigm.

The study procedure was as follows: (1) initial resting-state (RS1) scanning, (2) procedure of fear learning (FL) out of scanner (Pavlovian fear conditioning), (3) fear extinction (FE) scanning, and (4) second resting-state (RS2) scanning after FE. The time between the RS1 and FE sessions was ~45 min and between the FE and RS2, 1–2 min. The scanner parameters for the RS1, FE, and RS2 were the same, with equal session durations of 10 min (Figure 1). During the resting-state scanning, participants were asked to remain calm with eyes closed and to try not to think purposefully. A full description of the experimental procedure can be found in our previous article (Martynova et al., 2020). In the current study, we concentrated on analyzing three sessions on the first day of scanning to capture the immediate changes in the LRTC.

### Fear Learning and Fear Extinction Procedures

To minimize the association of the MRI scanner with negative stimulation, the procedure of FL was conducted in a separate room in the behavioral laboratory. The training constituted the presentation of two pseudo-random sequences with a short break between them. For FL, we used a delayed fear-conditioning paradigm with partial negative reinforcement. It consisted of presenting three visual stimuli. A Type 3 (CS-) figure was always neutral. The other two figures (CS1+ and CS2+) had the reinforcement probabilities of 70 and 30%, respectively. The unconditional stimulus (US) was weak electrical current stimulation for 500 ms, which was presented immediately after the figure when a white screen appeared. The strength of the stimulation was selected individually to be a tolerable but painful stimulus. Before each stimulus, participants saw a fixation cross lasting 2 s. The duration of each conditional stimulus (CS) varied randomly from 4 to 8 s with a step of 2 s. The presentation of each CS was followed by a white screen for a random duration of 8–12 s with a step of 2 s.

The second sequence was the same as the first, except that the probabilities of reinforcement for the CS1+ and CS2+ were changed by 30 and 70%, respectively. The total duration of each FL block was 8 min 54 s.

During the FE session, the same stimuli were presented, but in a different pseudo-random order and with a more extended overall sequence (10 min) and without US. An FE session was held during fMRI scanning. During this FE session, volunteers were asked to expect the US, but with a different reinforcement rule than in the previous two sessions.

### fMRI Data Acquisition

The MRI data were collected from the National Research Center Kurchatov Institute (Moscow, Russia) using a 3T scanner (Magnetom Verio, Siemens, Germany) equipped with a 32-channel head coil. The anatomical images were collected using a T1-MPRAGE sequence: TR 1470 ms, TE 1.76 ms, FA 9°; 176 slices with a slice thickness of 1 mm, a slice gap of 0.5 mm, and a 320-mm field of view (FoV) with a matrix size of 320 × 320. Functional images (300 volumes) were collected using a T2^∗^-weighted echo-planar imaging (EPI) sequence having a GRAPPA acceleration factor equal to 4 and the following sequence parameters: TR 2,000 ms, TE 20 ms, FA 90°; 42 slices acquired in interleaved order and having a slice thickness of 2 mm, a slice gap of 0.6 mm, and a 200-mm FoV with an acquisition matrix of 98 × 98. In addition, to reduce the spatial distortion of the EPI, the magnitude and phase images were acquired using a field-map algorithm that had the following parameters: TR 468 ms, TE1 4.92 ms, TE2 7.38 ms, FA 60°, 42 slices, and a 200-mm FOV. The imaging sequences and their durations were equal for the RS1, FE, and RS2.

### fMRI Preprocessing

The data regarding both the resting-state and FE sessions were processed using MELODIC, a part of FSL (FMRIB’s Software Library^1^). The following preprocessing steps were applied: motion correction (MCFLIRT), slice-timing correction using Fourier-space time-series phase-shifting, non-brain removal (BET), spatial smoothing using a Gaussian kernel of FWHM 5 mm, grand-mean intensity normalization of the entire 4D dataset using a single multiplicative factor, high-pass temporal filtering with a removing of the linear trends (Gaussian-weighted, least-squares, straight-line fitting, with sigma = 50.0 s, which equals a cutoff of 0.01 Hz) (Jenkinson et al., 2002; Smith, 2002). The B0-distortion was removed during the inserted B0-unwrapping algorithm. Registration of functional images to the individual anatomical and standard space MNI152 2 mm^3^ was conducted using FLIRT (Jenkinson and Smith, 2001; Jenkinson et al., 2002). Then, as part of the preprocessing step, independent component analysis (ICA) was performed using probabilistic ICA as implemented in MELODIC (v 3.14). For each participant fMRI signal, 38 independent components were extracted (Hyvarinen, 1999; Beckmann and Smith, 2004).

Next, additional de-noising of the data was conducted using FIX v1.068 [FMRIB’s ICA-based Xnoiseifier (Salimi-Khorshidi et al., 2014; Griffanti et al., 2014)] and ICA-based automatic removal of motion artifacts (ICA-AROMA) (Pruim et al., 2015a,b). First, the AROMA was applied to 15 datasets (five randomly chosen from each RS1, FE, and RS2 task group) in the classification regime to detect motion-related components. Then, the results were visually inspected according to recommendations (Griffanti et al., 2017) to detect additional artifact components, including cerebrospinal fluid (CSF) pulsation in the ventricles. Second, FIX was trained based on the preliminarily classified 15 datasets, and new automatic classification was applied to the remaining 54 sets (23 participants ^∗^ 3 scanning sessions) to detect noisy components, which were filtered out using FIX cleanup mode with the option to clean up the motion confounds (24 regressors). There were no significant within-subject differences in the number of removed components between sessions (mean 19.4 ± 5.06 ICs). Finally, the cleaned data were subjected to filtering to resting-state frequencies of 0.01–0.1 Hz using the “3dTproject” AFNI algorithm (Cox, 1996).

### Brain Parcellation

To extract time series for the LRTC analysis, the brain was parcellated into 246 areas according to the resting-state Brainnetome (BN) Atlas (Fan et al., 2016). Each mask was converted to each individual subject space using FLIRT FSL. The analysis found that region 94 in the BN Atlas, which corresponds to the right inferior temporal pole (BN-94-ITP-R), was absent in some persons who had brain sizes larger than the size of the FoV. Therefore, that mask was excluded from the subsequent analysis for all subjects, and only 245 areas were used.

In addition, the fear extinction network (FEN) from a meta-analysis (Fullana et al., 2018) and the task-related contrast (TRC) from our previous fear extinction study (Martynova et al., 2020) were used as regions of interest (ROIs). We took only 17 ROIs from the FEN (see Supplementary Table 1), which overlapped with those from the BN Atlas but were not located in the brainstem or the cerebellum. The same procedure was performed for the selection of TRC ROIs (11 clusters) (Supplementary Table 1). An averaged, normalized time course of BOLD response was extracted from each ROI.

### Analysis of Variance and Long-Range Temporal Correlations

The variance (σ^2^) was calculated for each time series, except for ROI BN-94-ITP-R, using the *numpy.var* Python function.

We used detrended fluctuation analysis (DFA) to access the LRTC in the fMRI signal. This method enables the estimation of long-range temporal dependence in time series. To perform DFA analysis, a Python script was applied from the Nolds package to the time courses extracted from the ROIs (Schölzel, 2019^2^). First, the trend was removed from the signal using a process of mean subtraction from each time point. Then, the cumulative sum was calculated by summing all numbers in the series. In the next step, the signal was divided into equal-size parts called windows. Within each window, the detrending procedure was repeated, and the standard deviation was calculated. The fluctuation intensity was measured by averaging the standard deviation of all windows. Using the same algorithm, fluctuation intensities were obtained for various chosen window sizes. The *H* value was calculated as the trend slope of the function of fluctuation intensity means vs. the window sizes in logarithmic scale (Peng et al., 1994; Hardstone et al., 2012). We chose non-overlapped windows with sizes of 12, 15, 20, 25, and 30 TR, and trend fitting was accomplished using the least-squares method. The minimal and maximal window lengths were selected based on those in previous studies. The minimal size chosen was 12 because Tagliazucchi et al. (2013) reported a trend deviation from linearity if the size of the time windows selected was less than 10 TR volumes on low-frequency resting-state BOLD signals. The maximum size was equal to one-tenth of the signal length (in volumes), as was suggested by Hardstone et al. (2012). Our data also showed a trend decline from linearity on windows larger than 30 volumes.

Furthermore, we estimated the goodness of fit for each *H* to understand how well it described the data. The goodness of fit was calculated as the squared correlation coefficient (*R*^2^). The mean *R*^2^ for each mask was > 0.95, which means they described the data well.

### Statistical Analysis

Because our aim was to trace the changes in variance and the LRTC in the resting state after fear exposure, as a first step, we used a non-parametric Wilcoxon signed-rank test to detect ROIs that had statistically significant differences in *H* and variance between RS1 and RS2. Then, we used only these ROIs to compare pairwise *H* and variance in the rest- and FE task-related signals using a Wilcoxon test. The association among anxiety scores, *H*, and the variance of all ROIs was tested using Spearman’s correlation analysis. The false discovery rate (FDR) correction (Benjamini and Hochberg, 1995) for multiple comparisons was applied to all statistical tests (MATLAB Bioinformatics Toolbox function *mafdr*).

## Results

### Variance of the BOLD Signal

**FIGURE 2:**
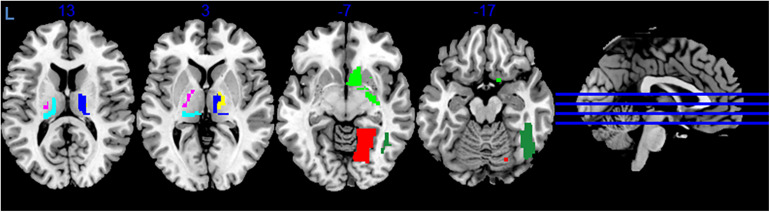
Areas exhibiting differences in variance of BOLD signal. Colors indicate different areas: BN-108 right fusiform gyrus, dark green; BN-196 right medio-ventral occipital cortex, red; BN-224 right nucleus accumbens, green; BN-233 left pre-motor thalamus, violet; BN-234 right pre-motor thalamus, yellow; BN-239 left posterior parietal thalamus, cyan; BN-246 right lateral pre-frontal thalamus, blue.

During variance analysis of the signal, we found 91 areas that had a difference between the RS1 and RS2, but the changes passed FDR correction in only 7 ROIs (Table 1 and Figure 2). Five of them were located in subcortical areas [right nucleus accumbens (BN-224-NAcc-R) and thalamic nuclei]. The other two were the right fusiform gyrus (BN-108-FuG-R) and the right medio-ventral occipital cortex (BN-196-MVOcC-R). All showed a difference in variance for a direct RS1–RS2 comparison (Table 1). Importantly, the variance of the BOLD signal in these ROIs demonstrated a steady increase from RS1 session to FE and RS2 session.

**TABLE 1:** Areas showing variance difference between sessions.

NN	Atlas number	Label ID	BA	Voxel size	L/R	Average variance (± σ)			Wilcoxon test					
									RS2-RS1		FE-RS1		RS2-FE	
	
						RS1	FE	RS2	*W*	*p*	*W*	*p*	*W*	*p*
1	108	Fusiform gyrus; FuG, A37lv, lateroventral area37	20/36/37	898	R	240.62 ± 135.65	239.49 ± 155.85	337.11 ± 177.77	23	0.0005*	138	1.0000	249	0.0007*
2	196	Medio ventral occipital cortex; MVOcC, rLinG, rostral lingual gyrus	18/19	858	R	299.04 ± 201.72	241.26 ± 118.16	404.38 ± 224.90	34	0.0016*	183	0.1711	246	0.0010*
3	224	Nucleus accumbens, NAC		427	R	180.99 ± 46.13	202.48 ± 58.49	229.59 ± 73.04	21	0.0004*	93	0.1711	80	0.0777
4	233	Thalamus; mPMtha, pre-motor thalamus		119	L	232.03 ± 43.21	268.96 ± 65.67	282.48 ± 71.22	27	0.0007*	52	0.0089*	173	0.2871
5	234	Thalamus; mPMtha, pre-motor thalamus		194	R	221.95 ± 33.29	234.59 ± 58.24	256.09 ± 51.56	33	0.0014*	108	0.3615	206	0.0386*
6	239	Thalamus; PPtha, posterior parietal thalamus		244	L	186.76 ± 48.16	188.21 ± 45.93	229.06 ± 72.45	28	0.0008*	122	0.6265	225	0.0081*
7	246	Thalamus; lPFtha, lateral pre-frontal thalamus		279	R	185.4 ± 40.80	197.99 ± 51.20	222.23 ± 61.58	41	0.0032*	102	0.2735	211	0.0264*

*Significant p-value (FDR-corrected).

### LRTCs of BOLD Signals

Of the 245 compared ROIs, we found 7 areas having substantial changes in the LRTC (H) between the pre-task and post-task resting states (*p*_*uncorr*_ < 0.05).

One area, the right superior frontal gyrus (BN-4-SFG-R), was excluded from further analysis, as its *R*^2^ values differed significantly during two RS sessions (*W* = 62, *p* = 0.0208), implying that they could not be statistically compared (Tagliazucchi et al., 2013) (Supplementary Table S2).

**FIGURE 3:**
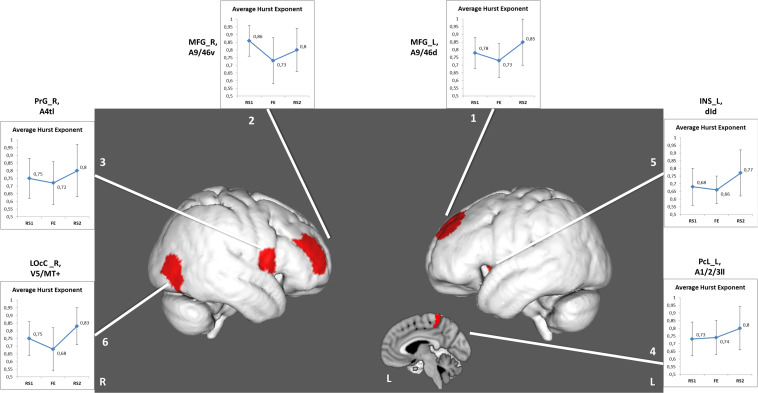
Regions depicting significant (p_*uncorr*_ < 0.05) changes in Hurst exponent. Numbered areas: 1, Middle frontal gyrus, left; 2, Middle frontal gyrus, right; 3, Precentral gyrus, right; 4, Paracentral lobule, left; 5, Insular gyrus, left; 6, Lateral occipital cortex, right.

The other six areas (Table 2 and Figure 3) showed prominent differences in *H* (Δ*H*) between RS1 and RS2. Only two of the areas exhibited a significant decrease in *H* during FE task performance: the right middle frontal gyrus (BN-22-MFG-R) and the right lateral occipital cortex (BN-202-LOcC-R). In contrast to other areas, which exhibited increased LRTC, BN-22-MFG-R demonstrated decreased LTRC of the BOLD signal in the RS2 compared to that in the initial RS1.

**TABLE 2:** Average Hurst exponent index in three sessions with comparison statistics.

NN	Atlas number	Label ID	BA	Voxel size	L/R	Average *H* (± σ)			Wilcoxon test					
									RS2-RS1		FE-RS1		RS2-FE	
	
						RS1	FE	RS2	*W*	*p*	*W*	*p*	*W*	*p*
1	15	Middle frontal gyrus; A9/46d, dorsal area 9/46	9, 10	1,077	L	0.78 ± 0.10	0.73 ± 0.11	0.85 ± 0.15	60	0.0177*	195	0.0830	232	0.0042*
2	22	Middle frontal gyrus; A9/46v, ventral area 9/46	10, 46	934	R	0.86 ± 0.10	0.73 ± 0.15	0.80 ± 0.14	208	0.0333*	240	0.0019*	204	0.0447*
3	62	Precentral gyrus; A4tl, area 4 (tongue and larynx region)	6/22/44	426	R	0.75 ± 0.13	0.72 ± 0.14	0.80 ± 0.17	66	0.0285*	166	0.3944	200	0.0593
4	65	Paracentral lobule; A1/2/3ll, area1/2/3 (lower limb region)	4/5/31	341	L	0.73 ± 0.11	0.74 ± 0.11	0.80 ± 0.14	71	0.0416*	127	0.7380	205	0.0416
5	173	Insular gyrus; dId, dorsal dysgranular insula	13	410	L	0.68 ± 0.12	0.66 ± 0.09	0.77 ± 0.15	58	0.0150*	139	0.9757	219	0.0138*
6	202	Lateral occipital cortex; V5/MT +, area V5/MT +	37, 19	808	R	0.75 ± 0.11	0.68 ± 0.14	0.83 ± 0.12	65	0.0264*	212	0.0244*	258	0.0003*

*Significant p-value (uncorrected).

### Variance and LRTC Changes in Task-Related Regions of Interest

**FIGURE 4:**
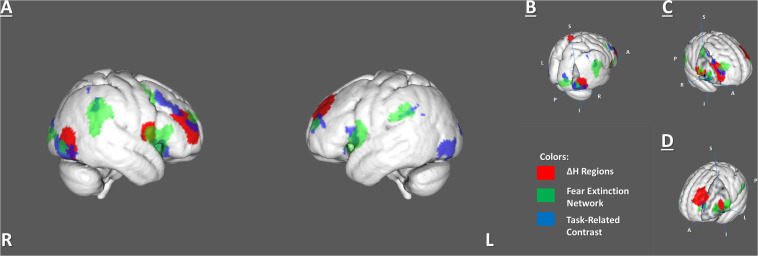
Overlapping areas among 6 identified ΔH regions, the fear extinction network, and task-related activity. **(A)** Whole-brain view of the overlaps. **(B)** Right lateral occipital cortex **(C)** Right inferior part of precentral and right middle frontal gyrus. **(D)** Left insula and middle frontal gyrus.

The *H* dynamic in the rest–task–rest paradigm was tested separately in the FEN and TRC clusters. There were no significant changes inside any of these areas. However, we found that five ROIs from the BN Atlas, that had significant Δ*H*, overlapped with the FEN clusters from the meta-analysis and the TRC clusters of the brain activations previously observed in FE (Supplementary Table 3 and Figure 4).

The BN-202-LOcC-R, BN-22-MFG-R, left middle frontal gyrus (BN-15-MFG-L), and right precentral gyrus (BN-62-PrG-R) areas overlapped with areas from both the FEN and TRC sets of clusters. The left insula (BN-173-INS-L) overlapped only with the FEN (Supplementary Table 3).

In addition, the *H* was calculated in each overlapping region of the BN ROIs with the FEN and the TRC separately. However, none of these overlaps had significant changes between sessions.

Furthermore, we checked the variance in ROIs, which exhibited Δ*H*. Some Δ*H* areas also showed changes in the variance: BN-62-PrG-R, left paracentral lobule (BN-65-PcL-L), BN-173-INS-L, and BN-202-LOcC-R (Table 3). In these, the variance insignificantly decreased during the task, but significantly increased during the post-task resting state.

**TABLE 3:** Variance of BOLD signal in brain areas with Δ*H.*

NN	Atlas number	Label ID	BA	Voxel size	L/R	Average variance (± σ)			Wilcoxon test					
									RS2-RS1		FE-RS1		RS2-FE	
	
						RS1	FE	RS2	*W*	*p*	*W*	*p*	*W*	*p*
1	62	Precentral gyrus; A4tl, area 4 (tongue and larynx region)	6/22/44	426	R	579.68 ± 422.77	645.58 ± 562.45	898.27 ± 803.62	51	0.0081*	117	0.5230	40	0.0029*
2	65	Paracentral lobule; A1/2/3ll, area 1/2/3 (lower limb region)	4/5/31	341	L	326.80 ± 308.65	269.71 ± 193.17	493.39 ± 481.44	59	0.0163*	127	0.7379	22	0.0004*
3	173	Insular gyrus; dId, dorsal dysgranular insula	13	410	L	280.21 ± 95.43	276.11 ± 92.84	340.56 ± 142.72	45	0.0047*	154	0.6265	220	0.0126*
4	202	Lateral occipital cortex; V5/MT +, area V5/MT +	37, 19	808	R	443.22 ± 350.67	356.51 ± 154.14	580.58 ± 298.73	61	0.0192*	151	0.6925	247	0.0009*

*Significant p-value (uncorrected).

In contrast to the *H*, the variance of the BOLD signal differed significantly in the FEN and TRC ROIs (Supplementary Table 4). Most ROIs exhibited a slight increase in variance during the task and RS2 (Supplementary Table 4).

We found no correlations of STAI scores with either variance or *H* of the BOLD signal in BN, FEN, and TRC ROIs or their overlaps.

## Discussion

In the present study, we investigated the variance and LRTC of brain activity measured using the BOLD signal in the rest–task–rest sequence. The process of extinguishing fear memories served as a task condition, as we were primarily interested in testing a hypothesis that short, negative emotional experiences during Pavlovian fear conditioning could influence the brain’s dynamics in the subsequent resting state after fear memory extinction. We compared the variance and the LRTC (measured using *H*) of the BOLD signal for 245 brain regions from the functional resting-state atlas (BN-ROIs) (Fan et al., 2016) in ROIs obtained from a meta-analysis of FE (FEN-ROIs) (Fullana et al., 2018) and in ROIs from an analysis of brain activation during the FE process in the same group of subjects (TRC-ROIs) (Martynova et al., 2020).

### Changes in the Variance of the BOLD Signal After Fear Learning and Extinction

Of the 245 BN-ROIs, we found 7 areas that had significant variance changes in dynamics during the rest–task–rest sequence. All areas exhibited the variance increase in RS2 relative to the RS1.

Increased nucleus accumbens (NAcc) variance has previously been shown to be associated with financial risk tasks (Samanez-Larkin et al., 2010). The NAcc also plays a role in fear conditioning and FE (Fullana et al., 2018). The increasing variance appeared consistent with prior literature showing a positive association between increasing variance and functional efficiency during active cognitive tasks (Garrett et al., 2012, 2018; Labrenz et al., 2019). The thalamic nuclei were associated with visual (BN-239-PPtha-L) and action execution (BN-233-PMtha-L) functions (Fan et al., 2016^3^), and their changes in variability seemed related to the experimental environment. A few other works have also shown changes in the variance of fMRI signals. A comparison of task-related and resting-state brain activity found increased dispersion of the BOLD signal in the inferior and dorsal prefrontal cortex and the default mode network areas (Garrett et al., 2012; Grady and Garrett, 2014). Higher variance was associated with higher cognitive performance (Garrett et al., 2013; Burzynska et al., 2015), which could indicate the level of adaptability and efficiency of neural systems because a more significant range of fluctuations enabled faster adaptation to various stimuli (Garrett et al., 2012). Other researchers have reported decreased variance in the visual cortex during visual discrimination tasks (Bianciardi et al., 2009) and in the areas of the resting-state networks when performing button-pressing tasks (He, 2011). These conflicting findings indicate that the dispersion of the BOLD signal may differ during different tasks depending on the experimental design.

### Changes in LRTC After Fear Learning and Extinction

We compared resting-state brain activity before and after the FE task and found 6 areas of 245 BN ROIs in which the LRTC of the BOLD signal changed but at uncorrected levels of significance.

In addition, we checked the possible differences in *H* between rest–task–rest sessions in the specific brain regions previously associated with fear processing, including the ROIs of the FEN taken from a meta-analysis of FE (Fullana et al., 2018) and areas of task-related brain activation during fear conditioning (TRC ROIs) obtained from the same group of subjects (Martynova et al., 2020). The LRTC of the BOLD signal did not change significantly. We assume that significant LRTC changes in the FEN and TRC ROIs might have been missing due to that our study focused on the resting-state data and the corresponding preprocessing pipelines, while masks of the FEN and TRC ROIs were obtained in the task-based fMRI designs and preprocessing based more on the task-induced neural activity rather than neural efficiency. However, we found that the most of regions from the BN Atlas, which demonstrated changes in *H*, overlapped with some FEN and TRC areas. This concurrence enables us to presume that the observed changes in *H* that reflected the LRTC were not accidental.

The LRTC of the BOLD signal decreased during the FE session. This finding is consistent with previous data reporting decreased LRTCs of fMRI signals during tasks (He, 2011; Ciuciu et al., 2012; Churchill et al., 2016). The decreased LRTC could be associated with neural activity underlining the more efficient processing of online information (He, 2011). Tasks that involved increased cognitive loads and increased novelty were accompanied by larger decreases in the LRTC (Churchill et al., 2016).

In the RS2, most areas, which had demonstrated decreased *H* during the task, experienced recovery of the *H* value to the initial levels of RS1. Importantly, the areas associated with FE showed not only recovery but also increased LRTC in the RS2, except the right middle frontal gyrus (BN-22-MFG-R). Barnes et al. (2009) traced the recovery of the *H* in the resting state after a memory task. The experiment’s design was similar to that of the current work, however, in Barnes et al. (2009), the second rest was twice as long as the initial rest and the task. The RS2 was divided into eight equal intervals, and the *H* was calculated for each. The signal complexity was gradually restored to its original level during a period of up to 15 to 18 min. In the present study, the *H* of the BOLD signal was averaged throughout a 10-min interval of RS, which might indicate the general complexity level of the neural activity after the FE task. It is also possible that a slight increase in the *H* index of the right middle frontal gyrus might reflect memory processing after the task, as this Brodmann area (BA46) is known to play a crucial role in working memory and attention (Pochon et al., 2002; Japee et al., 2015; Ueda et al., 2017).

The increased *H* in the other five areas can be explained by the relaxation of the neural activity and the possible consolidation of memory traces after the task. Duff et al. (2008) found increased low-frequency power spectral density during the RS2, and these changes occurred precisely in the areas associated with the motor task. In the present study, presumably, the increased *H* in the ROIs associated with the FEN resulted from internal neural adaptation and memory consolidation after the FE task. However, unlike in Duff et al. (2008), the *H* index was not adjusted for a specific frequency band but was based on the RS BOLD fluctuation filtered to low frequencies of 0.01–0.1 Hz. Our findings, in combination with Duff’s data, may indicate a possible interaction of scale-free processes with a change in a wide low-frequency range of the BOLD signal associated with neural activity during the task.

The BN ROIs, which had between-session differences in LRTC and variance, did not overlap. However, we also checked the signal variance in the ROIs with the changes in *H* between the RS1 and RS2. Few areas showed simultaneous increase of *H* and variance in post-task rest: the left paracentral lobule (BN-65-PcL-L), left insula (BN-173-INS-L), right precentral gyrus (BN-62-PrG-R), and right lateral occipital cortex (BN-202-LOcC-R). The decrease of variance and *H* in these areas during the FE was insignificant. Our findings are inconsistent with the data of B.J. He (2011), who reported a simultaneous decrease of the variance and LRTC in fMRI signals during task activation. The most significant changes were observed for the BOLD signal variance, showing a steady increase from RS1 to FE and, consequently, RS2. Importantly, we observed altered variance and *H* (at the uncorrected level of significance) in the resting-state brain activity after fear learning and extinction only in the specific brain regions related to the fear memory network (Feng et al., 2015; Fullana et al., 2018). These findings support our hypothesis that task-related activity during fear processing may modulate post-task spontaneous neural activity.

## Limitations

A few assumptions may limit the results of our research. First, neither did we have a control group that had similar scanning parameters nor did we compare the experimental group with a control, which should be done in future research. Fear-induced changes of BOLD dynamic may be confounded by regular changes over time, which can only be determined with a control group. The second limitation relates to the correction for multiple comparisons. Because we provided exploratory analysis without any directional hypothesis, we performed a pairwise comparison of *H* values in 245 ROIs. However, only 7 of the 245 showed a difference at the uncorrected *p*-value level. We assume that the pattern was not random, as all these ROIs overlapped with areas from a meta-analysis of the FEN (Fullana et al., 2018). The third limitation is due to the choice to parcellate the brain into ROIs rather than conduct voxel-wise analysis. This ROI-based approach averaged the BOLD signal across the brain area, which could considerably smooth the variability and fractality of the brain’s dynamics. However, we assume that the neural activity in these ROIs is synergistic, as we used ROIs from the brain atlas built on the functional parcellation of resting-state fMRI signals (Fan et al., 2016). Finally, the validity of the obtained results should be tested in further research using other methods for estimation of scale-free properties such as wavelet domain-based multifractal analysis.

## Conclusion

Using DFA and variability analysis, we demonstrated the changes in the scale-free properties and variance of the BOLD signals during the resting states after fear learning and extinction compared to the initial baseline resting-state condition. The pattern of changes in the LTRC (*H*) overlapped with those in the FEN ROIs in the brain cortex We found decreased *H* during the FE task, which replicated a previous finding (He, 2011), but we also found post-task changes in *H* only in areas related to fear processing network. The decreased LRTC may serve as a marker of specific task-related brain and residual memory processing. As a different method of analyzing fluctuation, the variance provided an additional measure of brain functional efficiency. It significantly increased in areas related to the processing of visual and emotional information. Not all areas with session-dependent *H* showed simultaneous changes in variance. However, in the right precentral gyrus, left paracentral lobule, lateral occipital cortex, and left insula, both the *H* and the variance decreased during tasks and increased during post-task rests. Overall, our work shows that changes in the resting states after fear learning and extinction can be captured using not only linear methods but also non-linear ones, including the variance and fractality of brain dynamics.

## Data Availability Statement

The datasets generated for this study are available on request to the corresponding author.

## Ethics Statement

The studies involving human participants were reviewed and approved by the Ethic Committee of the Institute of Higher Nervous Activity and Neurophysiology of Russian Academy of Sciences. The patients/participants provided their written informed consent to participate in this study.

## Author Contributions

OM, AI, and AT conceived and designed the study. SK and AT performed the experiments. AT analyzed the data. AT and OM wrote the manuscript. All authors contributed to the article and approved the submitted version.

## Conflict of Interest

The authors declare that the research was conducted in the absence of any commercial or financial relationships that could be construed as a potential conflict of interest.

## Footnotes

1 www.fmrib.ox.ac.uk/fsl

2 https://pypi.org/project/nolds

3 http://atlas.brainnetome.org/bnatlas.html
