# Supplementary material for: Variance and Scale-Free Properties of Resting-State Blood Oxygenation Level-Dependent Signal After Fear Memory Acquisition and Extinction
Source: Front Hum Neurosci. 2020 Oct 9;14:509075. doi: 10.3389/fnhum.2020.509075 (PMC7581738; doi:10.3389/fnhum.2020.509075)
Supplement: Supplementary file 1 [file Table_1.DOCX]

Supplementary Material

Supplementary Table 1. Regions of interest taken from Fear Extinction Network (Fullana et al., 2018) and Task-Related Contrast (Martynova et al., 2020). For the fear extinction network, we choose the areas, which overlaps with any labels from Brainnetome Atlas.

| NN | Name (peak) | L/R | **Voxel** | peak MNI | | |
| --- | --- | --- | --- | --- | --- | --- |
|  |  |  | **size** |  |  |  |
| Fear Extinction Network | | | | | | |
| 1 | Inferior Frontal Gyrus, orbital part | L | 1039 | -26 | 22 | -20 |
| 2 | Insula | R | 1581 | 28 | 18 | -18 |
| 3 | Lenticular Nucleus, pallidum | L | 270 | -10 | -8 | -14 |
| 4 | Inferior Occipital Gyrus | R | 136 | 44 | -82 | -12 |
| 5 | Calcarine Fissure | R | 420 | 20 | -96 | -6 |
| 6 | Thalamus | R | 35 | 8 | -22 | -6 |
| 7 | Middle Temporal Gyrus | R | 520 | 56 | -48 | 0 |
| 8 | Thalamus | L | 22 | -6 | -20 | 0 |
| 9 | Anterior Cingulate Gyrus | R | 2019 | 10 | 38 | 8 |
| 10 | Middle Frontal Gyrus | L | 260 | -28 | 50 | 10 |
| 11 | Middle Frontal Gyrus | R | 188 | 32 | 48 | 18 |
| 12 | Superior Temporal Gyrus | L | 111 | -64 | -30 | 20 |
| 13 | Cingulate Gyrus | R | 44 | 4 | -28 | 26 |
| 14 | Precuneus | L | 79 | -14 | -68 | 30 |
| 15 | Precental Gyrus | R | 247 | 42 | 2 | 34 |
| 16 | Cuneus | R | 27 | 16 | -68 | 38 |
| 17 | Precental Gyrus | L | 17 | -32 | -6 | 44 |
| Task-Related Contrast | | | | | | |
| 1 | Lingual Gyrus | L | 114 | -10 | -84 | -22 |
| 2 | Inferior Occipital Gyrus | L | 312 | -38 | -86 | -18 |
| 3 | Fusiform Gyrus | R | 566 | 46 | -72 | -18 |
| 4 | Inferior Frontal Gyrus, orbital part | R | 177 | 48 | 24 | -14 |
| 5 | Inferior Frontal Gyrus, opercular part | L | 128 | -52 | 18 | -10 |
| 6 | Middle Occipital Gyrus | L | 112 | -20 | -94 | 2 |
| 7 | Middle Frontal Gyrus | L | 100 | -36 | 52 | 8 |
| 8 | Middle Frontal Gyrus | R | 79 | 44 | 54 | 10 |
| 9 | Supramarginal Gyrus | L | 79 | -62 | -34 | 24 |
| 10 | Middle Frontal Gyrus | R | 191 | 48 | 30 | 28 |
| 11 | Supramarginal Gyrus | R | 162 | 58 | -46 | 30 |

Supplementary Table 2. The average Hurst exponent index in the excluded from analysis areas in three sessions with Wilcoxon-statistics and Friedman test.

| **NN** | **Atlas number** | **Label ID** | **BA** | **Voxel size** | **L/R** | **Average H** | | | **Wilcoxon test** | | | | | |
| --- | --- | --- | --- | --- | --- | --- | --- | --- | --- | --- | --- | --- | --- | --- |
|  |  |  |  |  |  |  |  |  | **RS1-RS2** | | **RS1-FE** | | **RS2-FE** | |
|  |  |  |  |  |  | **RS1** | **FE** | **RS2** | **W** | ***p*** | **W** | ***p*** | **W** | ***p*** |
| 1 | 4 | Superior Frontal Gyrus; A8dl, dorsolateral area 8 | 6/8 | 732 | R | 0.76 ± 0.14 | 0.70 ± 0.13 | 0.84 ± 0.11 | 58 | 0.0150 | 185 | 0.1529 | 253 | 0.0005 |

Supplementary Table 3. The relevance between Brainetome Atlas ROIs, which showed ΔH, with other areas. In the brackets, the areas are shown, which do not have a direct intersection with Brainnetome Atlas but located nearby.

| Brainnetome Atlas | | | Fear Extinction Network | | | Task-Related Contrast | | |
| --- | --- | --- | --- | --- | --- | --- | --- | --- |
| 15 | Middle Frontal Gyrus; A9/46d, dorsal area 9/46 | L | 10 | Middle Frontal Gyrus | L | 7 | Middle Frontal Gyrus | L |
| 22 | Middle Frontal Gyrus; A9/46v, ventral area 9/46 | R | 11 | Middle Frontal Gyrus | R | 8; 10 | Middle Frontal Gyrus | R |
| 62 | Precentral Gyrus; A4tl, area 4(tongue and larynx region) | R | 2 | Insula | R | 4 | Inferior Frontal Gyrus, orbital part | R |
| 173 | Insular Gyrus; dId, dorsal dysgranular insula | L | 1 | Inferior Frontal Gyrus, orbital part | L | (5) | Inferior Frontal Gyrus, opercular part | L |
| 202 | lateral Occipital Cortex; V5/MT+, area V5/MT+ | R | 4 | Inferior Occipital Gyrus | R | 3 | Fusiform Gyrus | R |

Supplementary Table 4.The variance statistics in the Fear Extinction Network and Task-Related Contrast areas. The table contains only ROIs, which shows significant variance difference in RS1-RS2 comparison.

| Type | **Label ID** | **BA** | **Voxel size** | **L/R** | **Average variance ± σ** | | | **Wilcoxon test** | | | | | |
| --- | --- | --- | --- | --- | --- | --- | --- | --- | --- | --- | --- | --- | --- |
|  |  |  |  |  |  |  |  | **RS1-RS2** | | **RS1-FE** | | **RS2-FE** | |
|  |  |  |  |  | **RS1** | **FE** | **RS2** | **W** | ***p*** | **W** | ***p*** | **W** | ***p*** |
| Fear Extinction Network | Inferior Frontal Gyrus, orbital part | 13/22/44/45/47 | 1039 | L | 324.35 ± 149.97 | 301.49 ± 134.80 | 436.15 ± 275.62 | 67 | **0.0308** | 164 | 0.4291 | 217 | 0.0163 |
|  | Insula | 13/44/45/47 | 1581 | R | 282.31 ± 142.74 | 295.57 ± 187.83 | 416.07 ± 300.79 | 40 | **0.0029** | 138 | 1.0000 | 226 | 0.0074 |
|  | Inferior Occipital Gyrus | 19 | 136 | R | 606.81 ± 477.26 | 646.14 ± 349.01 | 816.40 ± 614.55 | 57 | **0.0138** | 99 | 0.2355 | 200 | 0.0593 |
|  | Calcarine Fissure | 17/18/19 | 420 | R | 672.40 ± 498.12 | 788.52 ± 378.51 | 959.16 ± 557.34 | 51 | **0.0081** | 104 | 0.3011 | 185 | 0.1529 |
|  | Thalamus |  | 35 | R | 621.30 ± 116.78 | 670.81 ± 172.16 | 705.53 ± 148.51 | 70 | **0.0386** | 89 | 0.1361 | 172 | 0.3011 |
|  | Middle Temporal Gyrus | 22/40 | 520 | R | 418.53 ± 336.75 | 391.08 ± 218.44 | 536.66 ± 378.66 | 54 | **0.0106** | 123 | 0.6482 | 214 | 0.0208 |
|  | Thalamus |  | 22 | L | 760.29 ± 151.49 | 778.45 ± 177.01 | 880.09 ± 222.02 | 58 | **0.0150** | 127 | 0.7380 | 201 | 0.0553 |
|  | Precuneus | 7 | 79 | L | 489.15 ± 324.68 | 584.43 ± 542.22 | 586.74 ± 380.05 | 67 | **0.0308** | 67 | 0.0308 | 169 | 0.3458 |
|  | Cuneus | 7 | 27 | R | 688.49 ± 532.04 | 690.87 ± 312.20 | 839.83 ± 535.97 | 66 | **0.0285** | 122 | 0.6265 | 181 | 0.1909 |
| Task-Related Contrast | Lingual Gyrus | 18 | 114 | L | 757.43 ± 638.74 | 834.65 ± 609.81 | 1081.56 ± 804.46 | 50 | **0.0074** | 128 | 0.7610 | 205 | 0.0416 |
|  | Inferior Occipital Gyrus | 18/19 | 312 | L | 544.97 ± 417.50 | 789.42 ± 557.98 | 820.92 ± 592.55 | 64 | **0.0244** | 73 | 0.0480 | 141 | 0.9273 |
|  | Fusiform Gyrus | 18/19/37 | 566 | R | 536.50 ± 347.04 | 636.99 ± 300.83 | 793.44 ± 485.59 | 39 | **0.0026** | 97 | 0.2124 | 198 | 0.0680 |
|  | Inferior Frontal Gyrus, orbital part | 38/47 | 177 | R | 1995.53 ± 1544.79 | 2026.67 ± 1564.49 | 3016.79 ± 3007.62 | 51 | **0.0081** | 142 | 0.9032 | 233 | 0.0039 |
|  | Inferior Frontal Gyrus, opercular part | 44 | 128 | L | 1071.64 ± 599.67 | 1190.80 ± 731.25 | 1409.58 ± 937.60 | 52 | **0.0089** | 122 | 0.6265 | 206 | 0.0386 |
